# The role of astrocyte structural plasticity in regulating neural circuit function and behavior

**DOI:** 10.1002/glia.24191

**Published:** 2022-05-10

**Authors:** Oluwadamilola Lawal, Francesco Paolo Ulloa Severino, Cagla Eroglu

**Affiliations:** ^1^ Department of Cell Biology Duke University Medical Center Durham North Carolina USA; ^2^ Department of Neurobiology Duke University Medical Center Durham North Carolina USA; ^3^ Department of Neuroscience and Psychology Duke University Durham North Carolina USA; ^4^ Howard Hughes Medical Institute Duke University Durham North Carolina USA; ^5^ Duke Institute for Brain Sciences Durham North Carolina USA

**Keywords:** astrocytes, behavior, perisynaptic astrocyte processes, physiological states, synapses

## Abstract

Brain circuits undergo substantial structural changes during development, driven by the formation, stabilization, and elimination of synapses. Synaptic connections continue to undergo experience‐dependent structural rearrangements throughout life, which are postulated to underlie learning and memory. Astrocytes, a major glial cell type in the brain, are physically in contact with synaptic circuits through their structural ensheathment of synapses. Astrocytes strongly contribute to the remodeling of synaptic structures in healthy and diseased central nervous systems by regulating synaptic connectivity and behaviors. However, whether structural plasticity of astrocytes is involved in their critical functions at the synapse is unknown. This review will discuss the emerging evidence linking astrocytic structural plasticity to synaptic circuit remodeling and regulation of behaviors. Moreover, we will survey possible molecular and cellular mechanisms regulating the structural plasticity of astrocytes and their non‐cell‐autonomous effects on neuronal plasticity. Finally, we will discuss how astrocyte morphological changes in different physiological states and disease conditions contribute to neuronal circuit function and dysfunction.

## Main Points

- Astrocytes are highly complex cells which display structural plasticity. Here we review recent findings linking astrocyte structural plasticity to brain function and behavior.

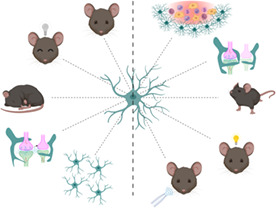

## 1 INTRODUCTION

Most synaptic connections in our brains are established during development and remodeled throughout life to adapt to changing circumstances (Molliver et al., 1973; Peter, 1979; Tierney & Nelson, 2009). Indeed, synapses undergo functional and structural changes, influenced by hardwired genetic plans, environmental factors, and experiences (Citri & Malenka, 2008; Ho et al., 2011; Mansvelder et al., 2019). A large body of work in neuroscience has focused on understanding how synaptic circuits are formed and remodeled via neuronal communication (Lu et al., 2009). However, neuronal processes which build synapses are highly integrated within a network of astrocytes (Eroglu & Barres, 2010; Haydon & Nedergaard, 2015; Nagai et al., 2021; Perez‐Catalan et al., 2021; Ventura & Harris, 1999).

Astrocytes, a major glial cell type in the brain, are highly complex cells that infiltrate the surrounding neuronal processes and synapses, collectively referred to as the neuropil (Bushong et al., 2002). Importantly, astrocytes actively control neuronal function by instructing synapse formation, plasticity, and remodeling (Allen & Eroglu, 2017; Baldwin & Eroglu, 2017; Chung et al., 2015). These essential functions of astrocytes at the synapse are intimately linked to their complex morphology, which is evolutionarily conserved (Oberheim et al., 2006; Stork et al., 2014). Remarkably, as the brain size and complexity of neuronal networks increased, so did the size and elaboration of astrocytes (Oberheim et al., 2006, 2009). For example, a single mouse astrocyte can interact with 100,000 synapses, whereas a human astrocyte is around three times larger and interacts with 2 million synapses (Bushong et al., 2002).

The fine perisynaptic astrocyte processes (PAPs) physically contact and ensheath pre‐ and postsynaptic specializations to form a tripartite synapses (Araque et al., 1999). The close interaction of PAPs with synapses is critical for regulation of synapse function through several mechanisms, including but not limited to gliotransmitter release and extracellular glutamate clearance (Allen, 2014; Chung et al., 2015). Furthermore, astrocytes undergo both gross and fine scale structural changes that may be playing important roles in neural circuit physiology and animal behavior (Allen, 2014; Arizono et al., 2021; Bernardinelli, Muller, & Nikonenko, 2014; Kleim et al., 2007; Santello et al., 2019). Gross morphological changes in astrocytes are also a hallmark of reactivity seen in many neurological diseases (Schiweck et al., 2018; Zhou et al., 2019). In this review, will discuss our current understanding of both gross and fine scale astrocyte structural plasticity and they may direct neural circuit function in health and disease.

## 2 EXPERIENCE‐DEPENDENT ASTROCYTE STRUCTURAL PLASTICITY

### 2.1 Sensory experiences strongly impact astrocyte morphological complexity

Synaptic circuits undergo substantial structural changes during development, which are strongly influenced by sensory experiences (Lendvai et al., 2000). Much of the research in this area utilized developing visual system circuits as their model. Visual experiences shape the connectivity of the brain circuits at the level of individual synapses by facilitating either their stabilization or elimination (Li et al., 2010; Ribic et al., 2019; Tropea et al., 2010). Astrocytes also play pivotal roles in synaptic remodeling in the developing visual system. Several astrocyte‐to‐neuron signaling pathways have been identified to take part in sensory experience‐dependent remodeling of synapses during critical periods of plasticity via the secretion of synapse‐modulating proteins (Blanco‐Suarez et al., 2018; Singh et al., 2016). Moreover, a recent study found that increasing the expression of connexin 30, a gap junction protein, causes closing of the critical period of plasticity in the mouse visual cortex by inhibiting an extracellular matrix‐degrading enzyme. The stabilization of the extracellular matrix, in turn, induces stabilization and maturation of inhibitory circuits and reduces visual circuit plasticity (Ribot et al., 2021). Importantly, manipulation of visual experiences, either in development or in adult, causes gene expression changes both in neurons and astrocytes of the visual cortex (Farhy‐Tselnicker et al., 2021; Hrvatin et al., 2018).

Sensory experiences not only affect astrocytic gene expression and extracellular matrix remodeling but also change astrocyte morphological complexity. There is an increase in astrocytic process elaboration and neuropil infiltration in the mouse visual cortex during development. The peak of astrocyte morphogenesis coincides with the period following eye‐opening, marking the onset of visual experience. This phenomenon occurs concurrently with increased neuronal glutamatergic synapses (Morel et al., 2014; Stogsdill et al., 2017). When astrocyte‐to‐neuron crosstalk is prevented, either by genetic deletion of the metabotropic glutamate receptor 5 (mGluR5) or the cell‐adhesion molecule neuroligin 2 (NL2) only in astrocytes, astrocytic elaboration is significantly reduced (Morel et al., 2014; Stogsdill et al., 2017). Interestingly, these astrocytic manipulations also impact synapse formation and synaptic activity, revealing the interdependent nature of astrocyte morphogenesis and synaptogenesis (Stogsdill et al., 2017).

**FIGURE 1:**
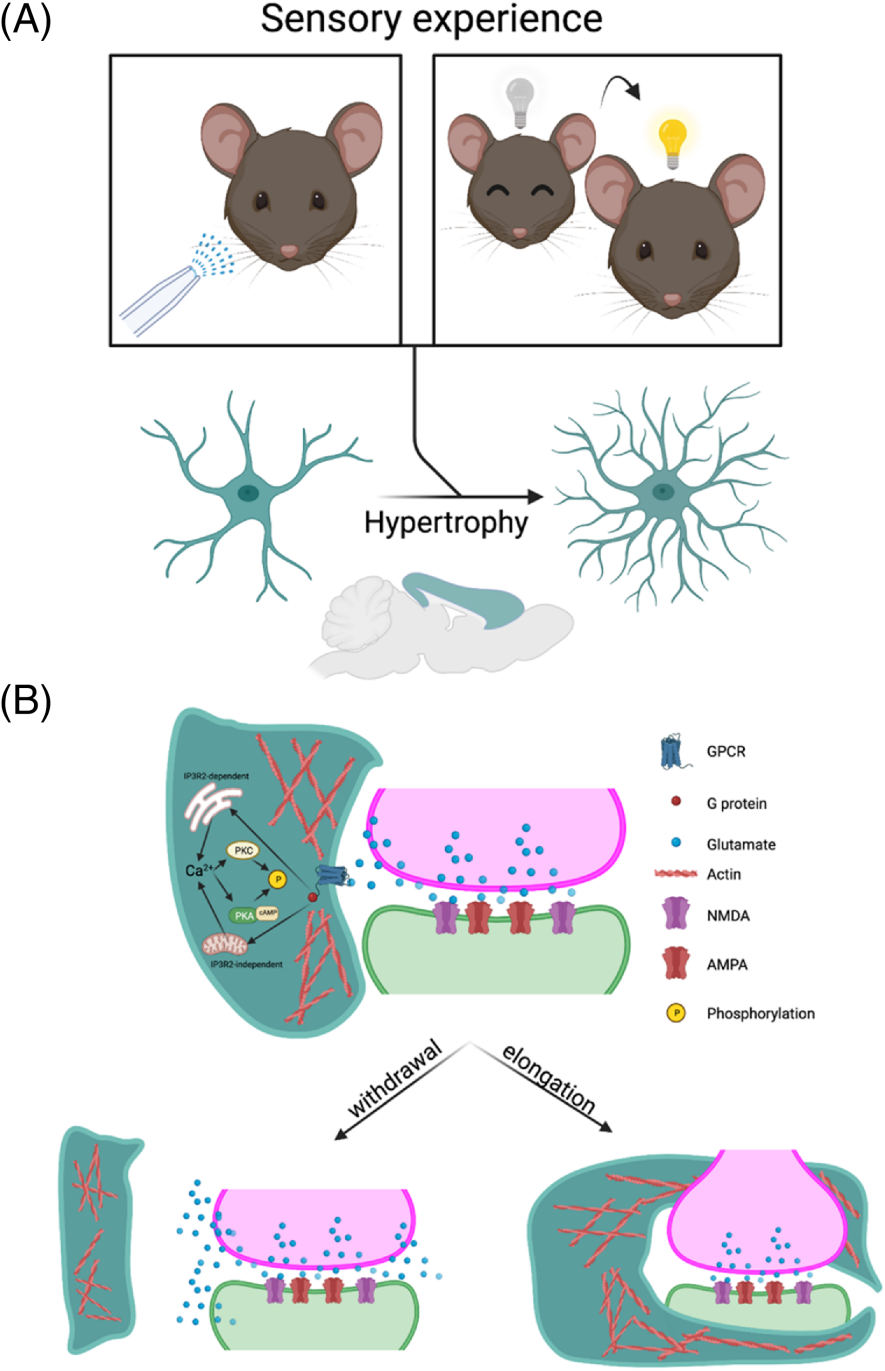
Sensory experience‐dependent astrocyte plasticity. (A) Sensory experience such as whisker stimulation and visual experience after eye‐opening drives cortical astrocyte hypertrophy and increase astrocyte process elaboration. (B) The activation GPCRs induces the release of Ca^2+^ in PAPs from intracellular stores such as the ER and mitochondria. Increased Ca^2+^ activity can trigger phosphorylation events that cause cytoskeletal reorganization and facilitate PAP elongation and withdrawal. PAP withdrawal may enhance the spillover of neurotransmitters such as glutamate, which may activate nearby synapses to promote LTP induction. Based on Bernardinelli, Randall, et al. (2014) and Perez‐Alvarez et al. (2014)

It is important to note that sensory‐dependent astrocyte plasticity is not restricted to development. In adult rats, monocular deprivation transiently increases the volume of the cortex on the contralateral side of the open eye. It is estimated that 71%–74% of the macroscopic tissue swelling is due to changes in astrocyte complexity with a 50% increase in territory volume (Figure 1a) (Schmidt et al., 2021). These changes in adult astrocyte morphology happen within a time course of days and weeks. Therefore, it is proposed to be a part of an experience‐dependent adaptation program of synaptic circuits.

Other forms of sensory experience also impact cortical astrocyte morphology. For example, whisker stimulation increases the expression levels of astrocytic glutamate transporters GLT‐1 and GLAST and astrocytic coverage of excitatory synapses in the mouse barrel cortex (Bernardinelli, Randall, et al., 2014; Genoud et al., 2006). Altogether, these findings link sensory experience‐dependent changes in synaptic circuits to astrocyte morphogenesis during development and adulthood. However, these studies also present new questions: How are experience‐dependent changes in astrocyte and neuronal morphology interlinked? Is it just a structural adaptation of astrocytes to the remodeling occurring at neuronal synapses, or do these structural changes in astrocytes have functional consequences on the synapses? Importantly, how dynamic are astrocyte‐neuron interactions at the synaptic sites?

One of the earliest reports about astrocyte process motility around synapses was made by Hirrlinger and colleagues (Hirrlinger et al., 2004). Utilizing an acute slice preparation, they described the structural changes of astrocytes around synapses within the brainstem through confocal and two‐photon microscopy. Two modes of motility were described: 1) gliding of thin lamellipodia‐like processes along the neuronal surface and 2) filopodia‐like structures extending from primary or secondary processes into the surrounding neuropil for several minutes. Since then, the anatomical descriptions of astrocytic processes around synapses have reached unprecedented details thanks to the development of super‐resolution microscopy techniques, like Stimulated Emission Depletion (STED) (Arizono et al., 2021; Arizono & Nägerl, 2022). Studies using STED showed that thin astrocytic processes are organized in reticular structures. In these structures, there are bulbous “nodes” which are estimated to be the predominant form of PAPs (Arizono et al., 2020). These nodes contain most of the spontaneous Ca^2+^ signals which are observed within astrocytes, showing that PAPs possess the machinery for Ca^2+^ signaling and activation of downstream pathways (Arizono et al., 2020). Indeed, several studies found correlations between Ca^2+^ transients within PAPs and experience‐dependent PAP motility. For example, time‐lapse imaging of PAPs in the sensory barrel cortex revealed that whisker stimulation increases PAP motility within 5–10 minutes from stimulus onset, resulting in an increased spine coverage by astrocytes and higher spine stability (Figure 1a). Similar results were also obtained by inducing long‐term synaptic potentiation (LTP), (Bernardinelli, Randall, et al., 2014; Perez‐Alvarez et al., 2014). LTP and long‐term depression (LTD) of synapses are two widely accepted cellular correlates of learning and memory. These cellular processes cause structural and functional changes at synapses, such as spine enlargement/shrinkage and surface delivery of neurotransmitter receptors (Collingridge et al., 2010; Fukazawa et al., 2003; Harris, 2020). It is interesting that astrocyte structure also responds to neuronal LTP. PAP motility is mediated by mGluRs, whose activation induces Ca^2+^ elevation in the PAPs. Applying a mGluR antagonist, an astrocyte‐specific calcium chelator (BAPTA‐AM), or knocking out the IP3‐receptor type 2 (IP3R2) blocked Ca^2+^ activity in PAPs and decreased their motility. On the contrary, the induction of Ca^2+^ fluctuations by activation of exogenous Gq‐coupled receptors caused an increase in PAP motility (Bernardinelli, Randall, et al., 2014; Perez‐Alvarez et al., 2014).

What is the molecular link between Ca^2+^ transients and motility of PAPs? One possible mechanism is protein phosphorylation, which is known to induce cytoskeletal reorganization and focal adhesion molecule turnover (Giannone & Sheetz, 2006; Lavialle et al., 2011; Niwa et al., 2002; Webb et al., 2004). Neuronal LTP was shown to drive PAP withdrawal through a cellular mechanism that involves the Na^+^‐K^+^‐2Cl^−^ cotransporter (NKCC1)‐cofilin‐1 pathway. NKCC1 induces the phosphorylation of cofilin‐1, which regulates actin polymerization. When phosphorylation of cofilin‐1 was inhibited, so was LTP‐induced PAP shrinkage (Henneberger et al., 2020). However, the mechanisms activating NKCC1 during LTP are not known. Protein phosphorylation events can be initiated by the interaction between a neurotransmitter/neuromodulator (e.g., glutamate) and a G‐protein coupled receptor (GPCR, e.g., mGluR) on the PAP membranes. GPCR‐mediated secondary messengers can trigger kinase activity. In the suprachiasmatic nucleus, such a mechanism occurs. It has been observed that the phosphorylated form of the actin‐binding protein, ezrin, is compartmentalized to the PAPs together with mGluR3 and mGluR5. The application of siRNA or dominant‐negative ezrin inhibits PAP motility (Lavialle et al., 2011). However, a direct link between astrocytic calcium transients in PAPs and phosphorylation of cytoskeleton‐binding proteins have not been shown.

Protein phosphorylation in astrocytes upon Ca^2+^ fluctuations could be IP3R2‐dependent or independent. The most studied form of Ca^2+^ activity in PAPs is the IP3R2‐dependent calcium release. This mechanism is triggered by GPCR activation and induces Ca^2+^ release from the endoplasmic reticulum (ER) (Srinivasan et al., 2015). IP3R2‐dependent Ca^2+^ release activates the protein kinase C (PKC) which could cause cytoskeletal rearrangements. Some Ca^2+^ transients in PAPs are IP3R2‐independent (Sherwood et al., 2017; Stobart et al., 2018). Interestingly, IP3R2‐independent Ca^2+^ fluctuations are detected in the juxtaposition of mitochondria location within the fine astrocytic processes (Agarwal et al., 2017). Therefore, IP3R2‐independent Ca^2+^ transients could indicate mitochondrial activity within PAPs. Mitochondria is necessary to supply ATP/GTP as part of the Ca^2+^‐calmodulin phosphorylation cascade and may be necessary to fuel PAP motility. The presence of these two types of Ca^2+^ activity indicates that different intracellular pathways mediate cytoskeletal changes and regulate PAP movement in astrocytes (Figure 1b). Alternatively, protein phosphorylation is the upstream controller of both Ca^2+^ transients and cytoskeletal changes. Indeed, cyclic AMP (cAMP), a secondary messenger mediating the activation of protein kinase A (PKA), modulates a subgroup of Ca^2+^ oscillations during astrocyte hypertrophy (Ujita et al., 2017). Even though these PKA‐dependent Ca^2+^ fluctuations and morphological changes were interpreted as a hallmark of reactive astrocytes, these signaling events could also occur in other non‐pathological circumstances and modulate astrocyte structural plasticity. In agreement with such a possibility, *in vitro* experiments showed that an analog of the cAMP can induce actin filament formation and the emergence of astrocytic processes (Baorto et al., 1992). Several other cellular pathways involving, for example, neurotrophin receptors and Rho GTPases (i.e. RhoA, Rac1, and Cdc42) have been shown to play roles in astrocyte morphogenesis and morphological changes (Holt et al., 2019; Zeug et al., 2018). However, most of these studies were conducted *in vitro* and *ex vivo* preparations. Nevertheless, they represent exciting starting points for future *in vivo* studies. Investigation of specific cellular pathways that control PAP dynamics and the causal links between PAP motility, astrocyte Ca^2+^ activity, and cytoskeletal rearrangements are poised to be fruitful future directions.

### 2.2 Astrocyte structural plasticity occurs during cognitive functions

One of the most fascinating cognitive functions of the brain is its ability to learn new skills and memorize environmental features and events. How astrocytes are involved in these brain functions has only recently been explored, with several studies reporting astrocytes' functional role in controlling cognition and behavior (Nagai et al., 2021; Oliveira et al., 2015; Santello et al., 2019). However, whether astrocytic structural changes are involved in regulating behavior is unclear. Here we will summarize some of the studies suggesting a possible link between astrocyte structure and cognitive function.

In the rodent cerebral cortex, learning new motor skills is linked to gross astrocyte morphological changes (hypertrophy), which increases the entire cell volume and the number of branches. However, such structural changes are not observed when mice simply repeat what they have already learned (Kleim et al., 2007). These findings suggested that astrocyte structural changes could occur under specific circumstances that induce synaptic plasticity. Therefore, to investigate behavioral consequences of the astrocyte structural changes, researchers focused on linking astrocyte structural plasticity to LTP/LTD, learning, and memory formation. One of the earliest descriptions of astrocytes' structural response to LTP was reported in the rodent hippocampus by Wenzel and colleagues (Wenzel et al., 1991). Astrocytes significantly increase their ramification and coverage of synapses 8 h following LTP induction. These observations have been confirmed and extended by another study showing that LTP induces enlargement of spine volume and increased coverage of pre‐ and post‐synaptic structures by astrocytes in an NMDA‐dependent manner (Lushnikova et al., 2009). These studies based their structural analyses utilizing transmission electron microscopy (TEM). The chemical fixation process involved in this technique might affect the morphology of both synapses and PAPs (Korogod et al., 2015), signifying the need for other methods, such as expansion microscopy and super resolution microscopy, to validate and interpret TEM studies in the future.

**FIGURE 2:**
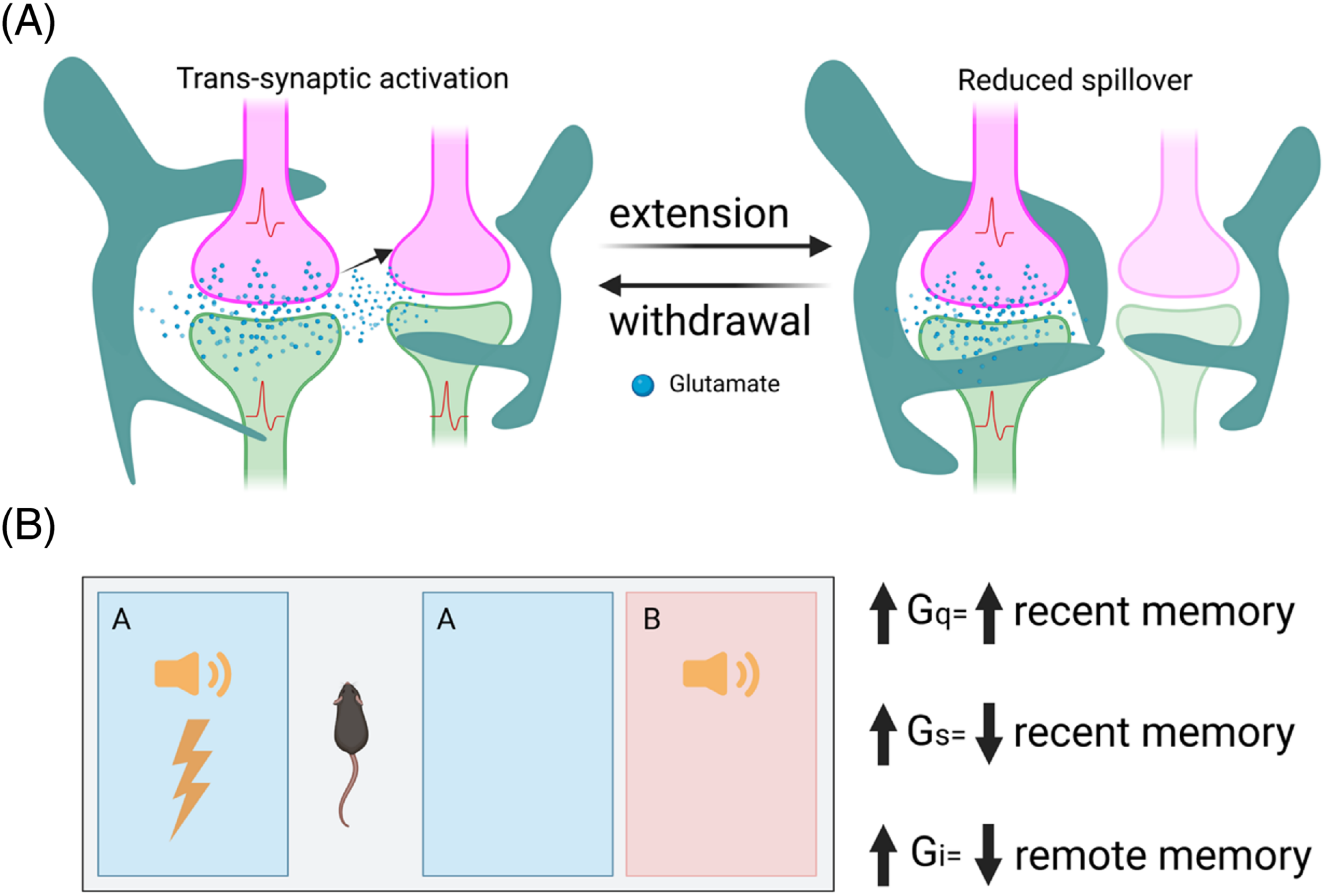
A possible link between astrocyte structural plasticity and GPCR activation during learning and memory formation. (A) Astrocytes processes may regulate neurotransmitter diffusion within the extracellular space through synapse coverage. By ensheathing synapses, astrocyte processes may prevent glutamate spillover and prevent trans‐synaptic activation, which could facilitate LTP (Henneberger et al., 2020; Herde et al., 2020; Ventura & Harris, 1999; Zheng et al., 2008). (B) During fear conditioning studies in which mice learn to associate a tone with a foot shock, activated GPCRs have different effects on memory formation. Gq enhances, and Gs impairs the formation of recent memories. However, the activation of Gi reduces remote memory recall (Adamsky et al., 2018; Kol et al., 2020; Orr et al., 2015).

Because PAPs and synapses are in close contact LTP‐induced structural changes in neurons are likely to impact astrocyte morphology. In agreement with this, neuronal LTP triggers dynamic extension and retraction of PAPs and neuronal spines until a new stable configuration is established (Bernardinelli, Randall, et al., 2014; Haber et al., 2006; Perez‐Alvarez et al., 2014). However, if and how astrocytic structural changes regulate LTP is still unclear. One proposed mechanism involves regulation of neurotransmitter diffusion via modulation of astrocytic coverage of synapses. For instance, glutamate release in the hippocampus and dopamine release in the striatum can activate nearby synapses through extrasynaptic diffusion of neurotransmitters (Rice & Cragg, 2008; Rusakov & Kullmann, 1998). This mechanism can result in co‐activation of nearby synapses thus facilitate the establishment of LTP. Experimental and modeling studies of extracellular diffusion of glutamate showed that astrocytes, through extension or withdrawal of their processes from synapses, control the rate of diffusion of the neurotransmitters to nearby synapses (Figure 2a) (Gavrilov et al., 2018; Kinney et al., 2013; McCauley et al., 2020; Ventura & Harris, 1999; Zheng et al., 2008). This mechanism is dependent on spine/synapse size. Indeed, smaller spines show a higher coverage by astrocytic processes than larger ones (Herde et al., 2020; Medvedev et al., 2014; Witcher et al., 2007, 2010), making the latter more protected by extrasynaptic glutamate diffusion because the glutamate uptake by the astrocytic glutamate transporter GLT‐1 is more efficient (Herde et al., 2020).

Another mechanism, mediated by the NKCC1‐cofilin 1 pathway, enhances synaptic crosstalk upon LTP induction, by initiating the withdrawal of PAPs to allow glutamate spillover (Henneberger et al., 2020). This structural rearrangement permits the NMDAR‐dependent activation of neighboring synapses (Figure 2a). Such phenomena could have relevant repercussions not only on synaptic potentiation but also learning and memory processes (McCauley et al., 2020). For example, associative learning in the lateral amygdala (LA) induces synaptic plasticity and morphological changes. Serial section TEM reconstructions showed that, after fear conditioning, there are increased numbers of synapses without astrocytic coverage. This effect was specific for larger synapses; whereas, smaller synapses had higher astrocytic coverage (Ostroff et al., 2014). Moreover, activation of the Ras‐related C3 botulinum toxin substrate 1 (Rac‐1) in cultured astrocytes induces astrocyte structural changes, and in vivo, Rac‐1 activation in astrocytes within the basolateral amygdala (BLA) during fear‐conditioning attenuates fear memory formation (Liao et al., 2017). These findings suggest that astrocyte morphology not only changes with circuit activity and sensory‐motor experience but could also regulates synaptic potentiation and innate, reflexive behaviors.

Insights into the role of astrocytes in behavioral control and memory formation come from functional studies in the hippocampus and prefrontal cortex. These two brain areas are indirectly connected, but show highly synchronized activity that supports cognitive task performance and remote memory consolidation (Doron & Goshen, 2018; Gordon, 2011; Preston & Eichenbaum, 2013). It has been shown that blockage of D‐Serine, a NMDAR co‐agonist, secretion by astrocytes in a dominant negative SNARE (dnSNARE) mouse model triggers a critical desynchronization of theta oscillation between the hippocampus and the prefrontal cortex, which impair spatial and long‐term memory (Sardinha et al., 2017). This mechanism is possibly linked to the activation of astrocytic type‐1 cannabinoid receptors which are necessary to induce D‐Serine secretion to drive LTP and memory consolidation (Robin et al., 2018). Moreover, astrocyte‐specific expression of tetanus neurotoxin in the hippocampus significantly reduces the gamma oscillations induced by the cholinergic agonist carbachol (Lee et al., 2014).

Studies investigating fear‐conditioning based learning in mice found that increasing intracellular calcium in hippocampal astrocytes, through the activation of GPCRs, have different effects on memory formation. Using Designer Receptors Exclusively Activated by Designer Drugs (DREADDs), it was shown that Gq or Gs activation have opposing effects (enhancement and impairment, respectively) on recent memory formation (Adamsky et al., 2018; Orr et al., 2015). In contrast, Gi activation impairs remote memory recall (Kol et al., 2020) (Figure 2b). Similar impairments of remote memory formation have been observed in studies in which NMDAR‐dependent LTD was altered due to astrocyte depletion of p38a MAPK or IP3R2 in mice (Navarrete et al., 2019; Pinto‐Duarte et al., 2019). In addition, within the medial prefrontal cortex, an increase in astrocyte Ca^2+^ activity through optogenetic activation of a light‐gated GPCR, melanopsin, enhances cortical activity and improve performance, decision‐making and working memory (Mederos et al., 2019, 2021).

Although these studies point toward the role of astrocytes in the control of animal behavior, there is not yet enough evidence for a causal link between behavioral control and astrocytes' structural plasticity. Even the effect of manipulating astrocytic Rac‐1 on fear memory formation (Liao et al., 2017) could be independent of Rac1's role in regulating PAP morphology. Thus, further research is needed to uncover the mechanisms that underlie the relationship between astrocyte morphology and behavioral control.

## 3 PHYSIOLOGICAL STATES AFFECT ASTROCYTE STRUCTURAL PLASTICITY

In addition to sensory experiences and synaptic plasticity, physiological states such as lactation, dehydration, starvation, and sleep strongly affect astrocyte structure and synapse ensheathment. A well‐established example of a role for astrocyte structure in physiological states comes from studies in the murine paraventricular (PVN) and supraoptic (SON) nuclei of the hypothalamus. Substantial structural and functional plasticity happens in the PVN and SON of female mice which are lactating. These changes are driven by both neuron–neuron and neuron‐astrocytes contacts (Chapman et al., 1986). The synthesis and release of oxytocin (OT) in these brain regions are pivotal for regulating lactation, and OT secretion depends on the electrical activity of magnocellular neurons (Oliet & Bonfardin, 2010). Astrocyte PAPs are retracted in the SON of lactating or dehydrated rats to regulate neuronal activity (Boudaba et al., 2003; Oliet, 2002). Several mechanisms have been described to regulate the activity of magnocellular neurons via astrocytic structural changes: 1) Excess of presynaptic glutamate release causes PAP withdrawal during lactation. This retraction facilitates extracellular diffusion of glutamate, which acts on presynaptic mGluRs triggering a negative‐feedback process to reduce presynaptic glutamate release and OT activity (Oliet, 2002; Oliet et al., 2001; Oliet & Bonfardin, 2010). 2) PAP withdrawal, due to glutamate release, causes the activation of kainate receptors (KARs) on GABAergic presynaptic terminals. During lactation, KAR activation leads to the inhibition of GABAergic transmission. This disinhibition facilitates post‐synaptic activation even when glutamate release is reduced (Oliet, 2002; Oliet & Bonfardin, 2010). 3) Astrocytes inhibit neuronal activity by releasing taurine, a gliotransmitter that activates glycinergic receptors and induces membrane hyperpolarization. PAP withdrawal reduces taurine's effect on synapses (Oliet, 2002). 4) Proximity of PAPs to neurons in the SON facilitates post‐synaptic activation by releasing NMDAR co‐agonist D‐Serine (Oliet & Bonfardin, 2010; Panatier et al., 2006). Thus, PAP withdrawal during lactation impairs NMDAR activation. During lactation, all these mechanisms act as a filter to limit the influence of external factors on OT‐mediated neuromodulation and ensure that only the information about the need for milk production is passed through.

A recent study showed that within the central nucleus of the amygdala (CeA), a morphologically distinct sub‐population of OT receptor‐expressing astrocytes can communicate through gap junctions upon OT release to promote positive emotional states (Wahis et al., 2021). This mechanism causes an increase in astrocytic calcium activity likely triggering the release of D‐Serine. D‐Serine increases the excitability of the interneurons in the centrolateral nucleus, enhancing the inhibition within the centromedial nucleus of the amygdala (Wahis et al., 2021).

Starvation also has strong impacts on astrocyte morphology. In a model of physical activity‐based anorexia, brain volume is reduced primarily due to a reduction in the number of astrocytes and their processes in the cerebral cortex and the corpus callosum (Frintrop et al., 2019). These changes in astrocyte numbers and gross morphology were caused by starvation, because refeeding was enough to recover the glial fibrillary acidic protein (GFAP) positive area. Two other studies support these observations in which astrocyte morphological changes were linked to food intake and calorie‐restricted diet. In mice after only 12 h, a high‐fat diet can induce an increase in astrocyte elaboration in the solitary tract of the brainstem dorsal vagal complex (MacDonald et al., 2020). Whereas a calorie‐restricted diet increases synapse ensheathment by hippocampal astrocytes, limiting glutamate spillover and enhancing LTP (Popov et al., 2020). These studies indicate that astrocytes respond to physiological states like lactation, hunger, and thirst, strongly impacting the function of the neuroendocrine system. Further research is needed to address the causal relationship between the astrocytes' structural plasticity and their roles in controlling the functionality of distant organs and glands through the neuroendocrine system.

### 3.1 Circadian and sleep‐related astrocyte structural plasticity

In most mammals, circadian rhythm, which operates on a 24‐h period, regulates physiology and behavior (Reppert & Weaver, 2002). Neuronal populations in the suprachiasmatic nucleus (SCN) have primarily been studied and established to control the circadian clock in a cell‐autonomous manner (Brancaccio et al., 2014; Liu et al., 2007; Maywood et al., 2011). However, even pure astrocyte cultures rhythmically express clock genes, period circadian protein homolog 1 and 2 (*Per 1 and Per2*) in a 24‐h period (Prolo et al., 2005). Interestingly, in SCN astrocytes, the intermediate filament protein GFAP expression fluctuates with circadian rhythm (Monique & Servière, 1993; Santos et al., 2005). Several studies pointed out a role for astrocytes in SCN circadian function (Prosser et al., 1994; Shinohara et al., 2000; Van Den Pal et al., 1992). Astrocytes were also shown to actively participate in circadian pace‐making through an anti‐phasic Ca^2+^ activity complimentary to neuronal Ca^2+^ events *ex vivo* in SCN slices (Brancaccio et al., 2017). In rodent SCN astrocytes, Ca^2+^ activity peaks during circadian nighttime and when there is a phasic release of glutamate into the extracellular space. In contrast, neuronal Ca^2+^ activity in the SCN peaks during circadian daytime (Brancaccio et al., 2017). Interfering with astrocytic gliotransmitter release or pharmacological inhibition of NR2C subunit of the NMDARs in the dorsal SCN neurons suppressed circadian oscillations (Brancaccio et al., 2017). More recently, Brancaccio and colleagues also found that astrocyte‐neuron communication in the SCN controls the circadian rhythm via regulation of SCN neuron gene expression (Brancaccio et al., 2019).

The SCN is essential in controlling circadian rhythm across the whole CNS by coordinating the activity of subordinate circadian oscillators in other brain regions, such as the hippocampus (Guilding & Piggins, 2007). Several genes and proteins associated with synaptic excitability exhibit circadian fluctuations in the hippocampus (Barnes et al., 1977; Debski et al., 2020). For example, the expression of the clock gene, *Per2*, is significantly increased in the CA1 pyramidal cell layer (CA1‐PC), *stratum radiatum (s*.*r*.*)*, and the SCN in the dark (D) phase of the circadian rhythm compared to the light (L) phase (McCauley et al., 2020). Interestingly, McCauley and colleagues found hippocampal astrocytes to also undergo structural plasticity during the L and D phase of the circadian rhythm (McCauley et al., 2020). Coinciding with circadian dependent gene expression changes in CA1, astrocytic coverage of post‐synaptic densities declines during the D phase of the circadian cycle. Astrocytic clearance of extracellular glutamate is also slower during the D phase, which impacts the temporal summation of AMPA receptor‐mediated excitatory postsynaptic currents (EPSCs) (McCauley et al., 2020). These structural changes, combined with a reduction in the cell surface expression of NMDARs on CA1‐PCs, lead to a reduction in LTP at the Schaffer collateral synapses in the D phase (McCauley et al., 2020). This difference in LTP magnitude based on L and D circadian phases may impact cognitive processes that are sensitive to high‐frequency hippocampal activity (McCauley et al., 2020). Astrocyte structural plasticity also plays a role in facilitating LTP in the L phase; however, the precise mechanism is not yet clear and requires further studies.

**FIGURE 3:**
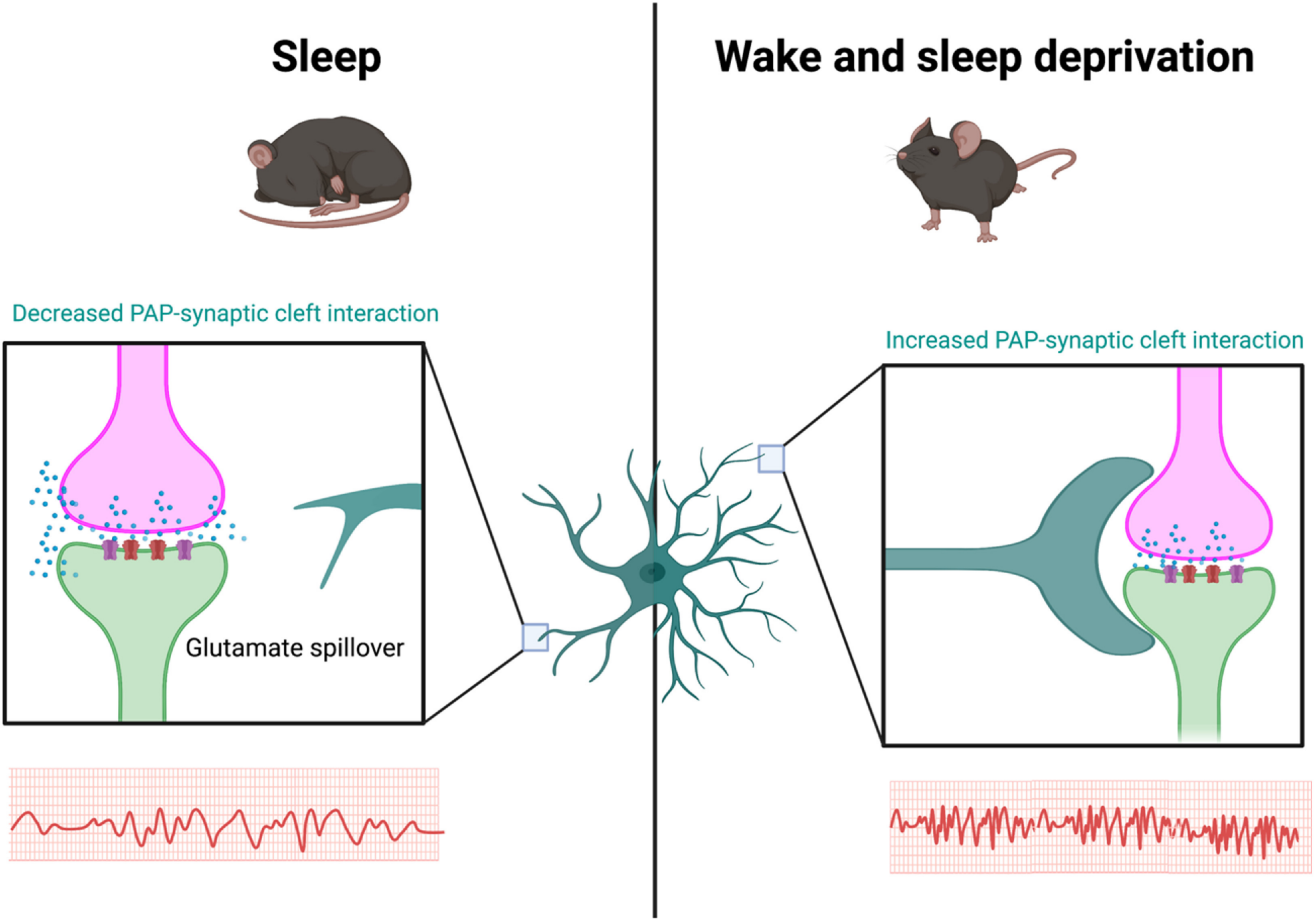
PAP plasticity during the sleep–wake cycle. Astrocyte processes are closer to the synaptic cleft during wake and sleep deprivation. In addition to increased synaptic contact, PAPs in chronic sleep‐restricted mice also increase their coverage of the neuropil (Not shown). In contrast, PAPs make less contact with the synaptic cleft during sleep (Bellesi et al., 2015). This is proposed to play a role in increased glutamate spillover during sleep. The resulting trans‐synaptic activation may play a role in the synchronization that is important for generating slow‐wave oscillations required for sleep and cognition.

A fundamental and essential physiological state that characterizes half of our daily life is sleep. The sleep–wake cycle is regulated by the circadian rhythm (Moore & Eichler, 1972; Saper et al., 2005). Albeit poorly understood, the mechanisms controlling sleep have also been traditionally viewed as primarily neuronal. However, this view is changing, because astrocytes strongly modulate sleep homeostasis through the release of adenosine, and the disruption of this astrocyte‐to‐synapse communication causes cognitive impairments, such as memory deficits (Florian et al., 2011; Halassa et al., 2009). Astrocyte structural plasticity has also long been proposed to play a critical role in regulating sleep. Over a century ago, Santiago Ramón y Cajal postulated that astrocytes extend their processes into the synaptic cleft to reduce synaptic transmission during sleep and retract their processes during wakefulness. Current evidence reveal changes in astrocyte morphology during sleep and wake, but not as dramatic as Cajal initially proposed. For example, cortical astrocytes undergo molecular and structural changes during the sleep–wake cycle. These changes are postulated to play a role in neuronal synchronization and glycogen turnover at synapses. During wake periods, mouse cortical astrocytes upregulate genes involved in cell process elongation and extend their processes closer to the synaptic cleft (Bellesi et al., 2015). Bellesi et al. performed serial block face scanning electron microscopical (SBF‐SEM) analyses of PAP dynamics in the layer II of the prefrontal cortex of wake, sleep, sleep‐deprived (SD), and chronic sleep‐restricted (CSR) mice. PAPs were closer to the synaptic cleft in the spontaneously wake mice during the D‐phase of the circadian rhythm. A similar configuration was also seen in the extended wake groups (SD and CSR) but during the L‐phase (Bellesi et al., 2015) (Figure 3). This finding suggests that wake increases PAP‐synapse interactions independent of the circadian clock. It is possible that the sleep–wake cycle impacts astrocyte structural plasticity in the cortex more readily compared to the hippocampus, where the effect of circadian rhythm dominates PAP‐synapse interactions (McCauley et al., 2020). The increased astrocyte coverage of the neuropil during the wake cycle might reflect the need for glutamate clearance (Bellesi et al., 2015).

Bellesi et al. found that the opposite occurs during sleep. Reduction of astrocyte processes around the synaptic cleft promote neuronal synchronization potentially through glutamate spillover (Bellesi et al., 2015). Indeed, glutamate transporter 1 (GLT‐1), which is essential for glutamate removal from synapses, is highly expressed in astrocyte processes in the neuropil (Minelli et al., 2001; Rothstein et al., 1994), and GLT‐1‐dependent glutamate clearance is modulated by neuronal activity (Armbruster et al., 2016). This model is also in line with the fact that an increase in extracellular glutamate, triggered using optogenetics, is sufficient to drive a switch to the slow‐oscillation‐dominated state in the mouse cortex, which is vital for sleep and memory (Poskanzer & Yuste, 2016). Astrocytes may also extend their processes into the neuropil during the wake cycle to position the glycogen granules, abundant within PAPs, closer to the synaptic cleft (Bellesi et al., 2018). This energy source could be significant in meeting the metabolic demands of neurons during wake (de Tredern et al., 2021; Díaz‐García et al., 2017). However, prolonged coverage of the synaptic cleft by the astrocytic processes through sleep deprivation can promote astrocytic phagocytosis of presynaptic components (Bellesi et al., 2017).

There is a possibility that the PAP dynamics during sleep are regulated by astrocyte Ca^2+^ transients. Bojarskaite et al. found that astrocytic Ca^2+^ transients occur with higher frequency within the neuropil during sleep–wake transitions (Bojarskaite et al., 2020). There is a reduction in overall astrocyte Ca^2+^ activity during sleep; however, Ca^2+^ transients during sleep are more frequent in the astrocytic processes than the soma (Bojarskaite et al., 2020). The Ca^2+^ signaling within PAPs may facilitate their retraction from the neuropil during sleep (Bellesi et al., 2015; Bernardinelli, Randall, et al., 2014; Perez‐Alvarez et al., 2014). Interestingly, astrocytic Ca^2+^ transients can also regulate non‐rapid eye movement (NREM) sleep features, which are important for memory consolidation (Vaidyanathan et al., 2021). Chemogenetic activation of astrocytic Gi‐GPCR to drive Ca^2+^ activity in astrocytes increases slow‐wave activity (SWA), an oscillatory pattern of cortical neural activity during NREM sleep. This Gi‐driven increase in SWA regulates sleep depth but not duration (Vaidyanathan et al., 2021). Surprisingly, activation of the Gq‐GPCR through chemogenetics suppressed Ca^2+^ transients in astrocytes and disrupted sleep–wake transitions, thereby leading to increased sleep duration (Vaidyanathan et al., 2021). However, it is still unclear whether astrocytic Gq‐signaling specifically regulates sleep–wake transitions or whether suppressing Ca^2+^ in astrocytes through other means would also produce a similar result. Furthermore, a link between Ca^2+^ transients and structural changes in astrocytic processes during sleep–wake cycles remains to be established.

## 4 ASTROCYTE STRUCTURAL PLASTICITY IN AGING AND DISEASE

**FIGURE 4:**
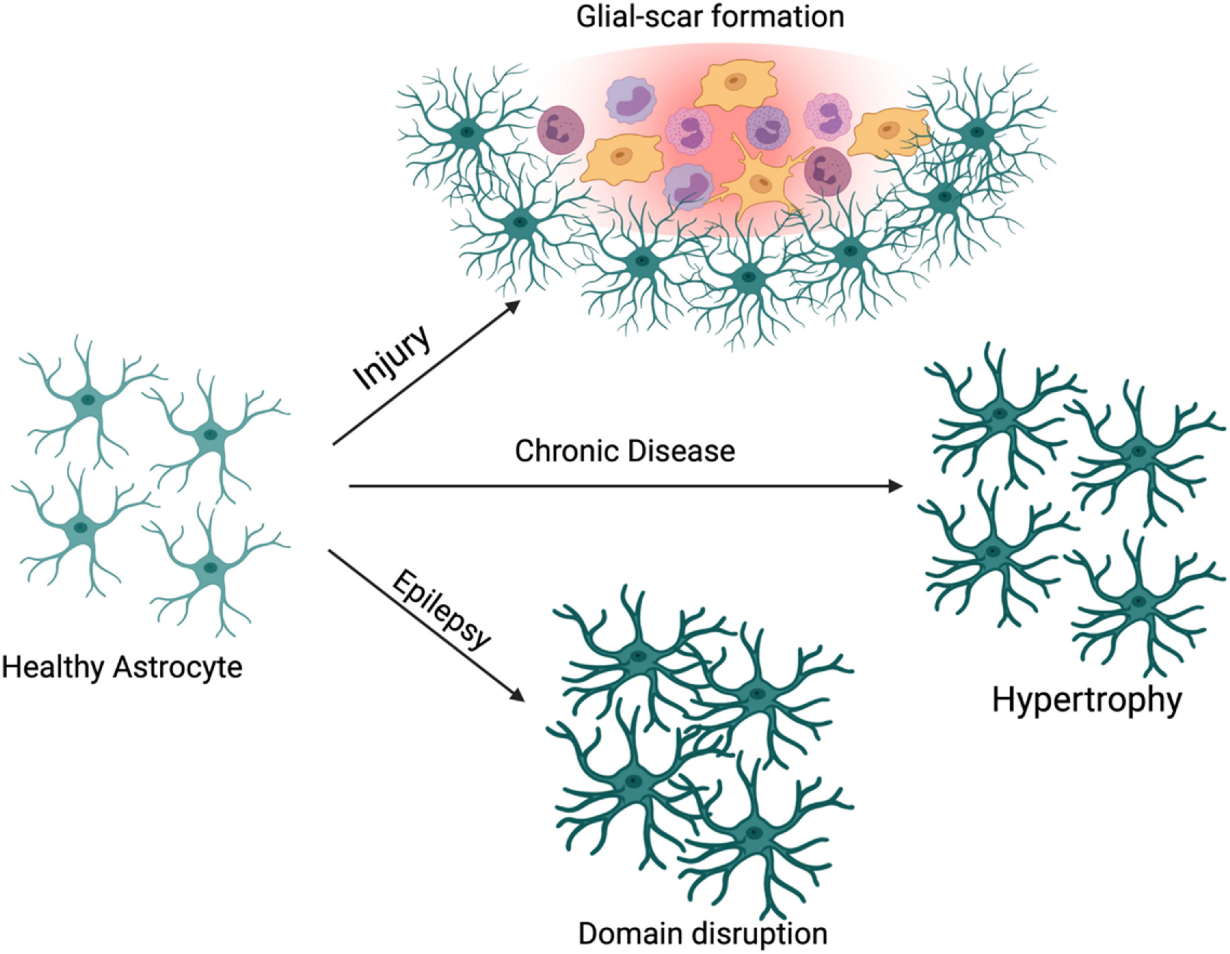
Injury and disease change astrocyte morphology. In a healthy brain, astrocytes extend their processes but maintain non‐overlapping domains. In contrast, astrocytes undergo astrogliosis during injury, which is characterized by hypertrophy and the formation of glia‐scar around the site of injury to protect healthy tissues around the injury site (Bush et al., 1999; Faulkner et al., 2004). Astrocytes have increased and thicker primary processes in chronic disease but still maintain their domains. However, the non‐overlapping domain structure is disrupted in epileptic brains (Oberheim et al., 2008).

Studies that evaluated astrocyte structural plasticity in aging, injury, and disease have primarily focused on astrogliosis, a term that describes the morphological, transcriptional, and functional changes associated with “reactive astrocytes” (Ben Haim et al., 2015; Guttenplan et al., 2020, 2021; Liddelow & Barres, 2017; Sofroniew, 2020; Zhou et al., 2019). The phenotypes that describe reactive astrocytes are diverse and dependent on the pathological context (Escartin et al., 2021; Liddelow & Barres, 2017). However, across all pathological conditions that lead to astrogliosis, there is a common theme: reactive astrocytes change their morphology, increase the expression of cytoskeletal proteins such as GFAP and vimentin, secrete inflammatory factors, and proliferate (Figure 4) (Ben Haim et al., 2015). A major morphological hallmark of reactive astrocytes is hypertrophy (Figure 4) (Zhou et al., 2019). Hypertrophic astrocytes have enlarged cell bodies and elongated major processes with increased thickness (Bardehle et al., 2013). These hypertrophic phenotypes are often characterized through the enhanced immunoreactivity of GFAP, an intermediate filament protein that is upregulated in reactive astrocytes (Wilhelmsson et al., 2004). GFAP and vimentin, another intermediate filament protein, play critical roles in astrocyte reactivity, glial‐scar formation, and hypertrophy (Liu et al., 2014; Pekny et al., 1999; Wilhelmsson et al., 2004). The absence of GFAP and vimentin significantly reduces astrocytic process hypertrophy (Wilhelmsson et al., 2004). Visualization of GFAP immunostaining in reactive astrocytes demonstrates cytoskeletal reorganization during astrogliosis but does not necessarily indicate cellular hypertrophy. Some studies addressing this knowledge gap have utilized cell‐filling dyes to investigate the actual three‐dimensional morphology of reactive astrocytes (Bardehle et al., 2013; Wilhelmsson et al., 2006). They found that in response to a lesion, astrocytes became reactive. The number and the thickness of the main processes protruding from the soma were increased. The total volume accessed by reactive astrocytes and their territorial domains remained unchanged even though GFAP‐labeling indicated elongated processes. This observation suggests that changes in GFAP immunoreactivity may not fully reflect the changes in astrocyte morphology. Live‐imaging of astrocytes in response to a cortical lesion demonstrated that while a subset of reactive astrocytes displayed the phenotype observed in Wilhelmsson et al. (2006), there were also two other subsets, one with elongated processes directed toward the lesion site, and another with proliferative markers, which are localized to juxtavascular space (Bardehle et al., 2013). Furthermore, in mouse models of epilepsy, cortical astrocytes lose their non‐overlapping domain organization due to astrogliosis (Oberheim et al., 2008) (Figure 4). All these observations suggest that the structural plasticity of reactive astrocytes is heterogeneous, prompting the question of the role of these different subsets in response to pathology and maintenance of homeostasis.

The functional consequences of astrocyte reactivity are complex. Astrogliosis has been shown to both exacerbate ongoing pathology and promote homeostasis. For example, proliferating and hypertrophic reactive astrocytes form a cellular barrier (also known as glial scar) around a traumatic injury site in the CNS to protect surrounding healthy tissues (Bush et al., 1999; Faulkner et al., 2004). This barrier limits the infiltration of inflammatory cells, decreasing tissue damage, and providing trophic support to regenerating axons (Figure 4) (Anderson et al., 2016; Bush et al., 1999; Faulkner et al., 2004; Sofroniew, 2009). Ablation of reactive astrocytes post‐injury in hippocampal CA1 exacerbated neuronal loss (Bush et al., 1999). This mechanism may be due to the increased sensitivity of CA1 pyramidal neurons to excitotoxicity as a result of disrupted glutamate uptake by astrocytes (Rothstein et al., 1996). In mice lacking GFAP and Vimentin (GFAP^−/−^Vim^−/−^), hypertrophy of astrocytic processes in response to injury was remarkably reduced compared to WT. This effect was accompanied by a more dramatic loss of synaptic proteins and axon degeneration in GFAP^−/−^Vim^−/−^ mice around the injury site (Wilhelmsson et al., 2004). However, 14‐days post‐injury, GFAP^−/−^Vim^−/−^ had completely restored the number of synaptic proteins to a level comparable to the uninjured side, while the WT experienced marginal recovery (Wilhelmsson et al., 2004). Taken together, these data suggest that astrocyte hypertrophy and formation of glial‐scar prevents neuronal and synapse damage shortly after injury but they may also limit synapse recovery and axon regeneration in the long term (Bush et al., 1999; Silver & Miller, 2004; Wilhelmsson et al., 2004). However, many questions remain regarding the mechanisms that define and drive astrocyte reactivity in different CNS pathologies and how structural changes in reactive astrocytes impact neuronal function.

Reactivity is also associated with massive alterations in gene expression profiles of astrocytes (Anderson et al., 2016; Hasel et al., 2021; Liddelow & Barres, 2017; Orre et al., 2014; Sofroniew, 2009; Zamanian et al., 2012). There is no consensus of genes altered in reactive astrocytes across all pathologies, indicating differences in mechanisms that drive different diseases. However, genes such as inwardly rectifying potassium channel subunit Kir4.1, and glutamate transporter GLT‐1, are downregulated or dysfunctional in reactive astrocytes (Nwaobi et al., 2016; Sheldon & Robinson, 2007). For example, in Huntington's disease (HD) mouse models, astrocytes reduce Kir4.1 expression, increasing extracellular potassium (K^+^) levels. The reconstitution of Kir4.1 expression was sufficient to decrease the hyperexcitability of striatal medium spiny neurons and improve HD‐associated motor deficit (Tong et al., 2014). Also, reduced GLT‐1 expression has been demonstrated in reactive astrocytes of the human neocortex following traumatic brain injury (Landeghem et al., 2006). Loss of astrocytic GLT‐1 may impair glutamate clearance, promote excitotoxicity, and cause neuronal death. Indeed, knockdown of GLT‐1 in astrocytes exacerbates neuronal damage in a rat model of traumatic brain injury and cerebral ischemia (Rao, Dogan, Bowen, et al., 2001; Rao, Dogan, Todd, et al., 2001). It is plausible that hypertrophy of reactive astrocytes in these pathological states is a compensatory mechanism to localize the essential channels and transporters next to synapses.

Aging is a common risk factor for many neurological diseases particularly neurodegeneration (Hou et al., 2019). Therefore, it is necessary to investigate astrocytic changes during aging as it may be protective or contribute to disease pathology. During aging, structural and transcriptional changes occur in astrocytes and these changes differ between brain regions (Boisvert et al., 2018; Clarke et al., 2018; O'Callaghan & Miller, 1991; Rodríguez et al., 2014). For example, in the striatum and hippocampus, astrocytes increase their expression of GFAP and become more hypertrophic with aging. In contrast, astrocytes in the entorhinal cortex are less hypertrophic in the aged mice (Bondi et al., 2021; Rodríguez et al., 2014). Interestingly, hippocampal and striatal but not cortical astrocytes upregulate genes which are also abundant in reactive astrocytes (Clarke et al., 2018). The increased expression of these reactive astrocyte markers in the striatum and hippocampus suggests that these brain regions are more vulnerable to pathology during aging. However, how these differences in astrocyte hypertrophy across brain regions contribute to circuit function and dysfunction during aging is still largely unknown.

One of the earliest pieces of evidence of astrocyte reactivity, as indicated by GFAP immunostaining, was in the brains of Alzheimer's disease (AD) patients (Bignami et al., 1972). Indeed, astrocytes near amyloid‐beta plaques increase their GFAP expression and become hypertrophic (Vijayan et al., 1991), and in some cases display altered water and potassium channel expression (Wilcock et al., 2009). A study by Jo et al. found plaque‐associated hypertrophic astrocytes to have aberrant GABA release in the dentate gyrus of AD mouse model (Jo et al., 2014). This dysfunction strongly inhibited synaptic transmission by decreasing spike probability at a specific synaptic connection within the hippocampus, resulting in a learning and memory deficit (Jo et al., 2014). Plaque‐associated hypertrophic astrocytes with extended processes may also engulf dystrophic neurites in the hippocampus of AD mouse model and AD patients (Gomez‐Arboledas et al., 2018). In certain AD mouse models, astrocytes within the hippocampus and the entorhinal cortex, which are not associated with plaques, undergo cytoskeletal atrophy (Olabarria et al., 2010). One study found that astrocyte hypertrophy and atrophy can be prevented through environmental enrichment in an AD mouse model (Beauquis et al., 2013). However, the mechanisms through which AD‐related astrocyte morphological changes occur and the impact of these changes on neuronal function and behavior are unknown and require further studies.

Morphological changes in astrocytes have also been implicated in other neurodegenerative diseases. For example, in post‐mortem brain tissues of patients, astrocytes bordering multiple sclerosis (MS) lesions are hypertrophic (Black et al., 2010). In the SOD1 transgenic mouse model of Amyotrophic Lateral Sclerosis (ALS), reactive and hypertrophic astrocytes upregulate GFAP expression in the spinal cord. Additionally, a subset of these astrocytes surrounding spinal motor neurons had unusual spheroid‐shaped cell bodies that were positive for the active form of caspase‐3, which would later cleave GFAP (Rossi et al., 2008). Post‐mortem tissues from Huntington's disease (HD) patients show increased hypertrophy in astrocytes which contain mutant huntingtin (mHtt) aggregates (Faideau et al., 2010). Interestingly, astrocyte reactivity in ALS and HD is linked to alteration in potassium channel expression and function (Kaiser et al., 2006; Tong et al., 2014), suggesting a potential target for therapeutics.

Though much focus has been on how pathology impacts astrocyte morphology, there is mounting evidence that impairment in astrocyte structural plasticity can causally disrupt circuit formation and function and drive disease pathogenesis. In Alexander disease (AxD), a heterozygous mutation in GFAP causes hypertrophy and accumulation of cytoplasmic protein inclusions called Rosenthal fibers within astrocytes (Messing et al., 2012). These astrocytes activate the mTOR pathway (Tang et al., 2008), display impaired gap junction coupling (Olabarria et al., 2015), and lose GLT‐1 (Tian et al., 2010). The loss of GLT‐1 in these hypertrophic astrocytes is proposed to be the driver of neuronal loss in AxD due to glutamate‐induced excitotoxicity (Tian et al., 2010).

Cell adhesion molecules which bridge PAPs and synapses are critical for the interdependent development and function of astrocytes and neurons. For example, loss of neuroligin family cell adhesion molecules impair astrocyte morphogenesis. Specifically, the depletion of astrocytic neuroligin‐2 has been demonstrated to not only reduce astrocyte morphological complexity but also alter neuronal excitation/inhibition balance (Stogsdill et al., 2017). Mutations in neuroligins and their interacting partners, neurexins, are implicated in schizophrenia (Sun et al., 2011). Interestingly, the expression of genes encoding for these adhesion molecules were downregulated in human induced pluripotent stem cell (hIPSC)‐derived astrocytes from schizophrenia patients which were grafted to wild‐type mouse brains (Windrem et al., 2017). Grafted patient astrocytes had impaired morphologies and caused neuronal dysfunction (Windrem et al., 2017). Another disease‐linked cell adhesion protein HepaCAM is highly enriched in astrocytes (Baldwin et al., 2021; Sofroniew, 2021). HepaCAM point mutations cause megalencephalic leukoencephalopathy with subcortical cysts (MLC), a disorder that presents with intellectual disability, autism and epilepsy in humans (López‐Hernández et al., 2011). Loss of HepaCAM function in astrocytes alone strongly impairs astrocyte morphogenesis by disrupting gap junction coupling. These changes in astrocytes are sufficient to strongly impact synaptic function by dysregulating excitatory and inhibitory synaptic strengths in the mouse cortex (Baldwin et al., 2021). Taken together these studies reveal the importance of bi‐directional structural and functional coupling between astrocyte and neuron networks in establishing and maintaining brain homeostasis.

## 5 CONCLUSIONS AND FUTURE DIRECTIONS

For a long time, astrocytes were thought to be the mere support cells for neurons and synapses. However, for the last three decades, neuroscientists began to recognize the importance of astrocytes in brain circuit formation and regulation. Still, the roles astrocytes play in information processing and behavioral control capabilities of brain circuits are largely unknown. This review summarizes some of the studies investigating the relationship between astrocytic structure and function, focusing on its impacts on neuronal activity and plasticity under physiological and disease states. A common theme underlying these findings is that, at synapses, astrocytes monitor, respond, and regulate glutamate release and post‐synaptic activity. Withdrawal and extension of PAPs in response to glutamate can enhance post‐synaptic responses, inhibit trans‐synaptic activation, and inhibit further glutamate release. However, how astrocyte structural plasticity change upon release of other neurotransmitters (such as GABA, dopamine, somatostatin, serotonin, acetylcholine, etc.) is not known. Are all astrocytes responding in the same way to neurotransmitters, or is there regional heterogeneity in their structural plasticity? These questions remain to be answered.

We also lack information about the specific pathways utilized to reorganize the cytoskeleton of PAPs, to regulate the release of gliotransmitters and synaptogenic factors, and to promote the uptake of ions and neurotransmitters from the extracellular space. Neurotransmitter mediated GPCR signaling in astrocytes could induce the observed structural changes. So far GPCR signaling within astrocytes were mostly performed by using overexpression of exogenous designer GPCRs to induce dramatic intracellular calcium elevations. The endogenous GPCR‐signaling within PAPs is poorly understood.Future experiments to understand the molecular pathways regulating astrocytes' structural and functional responses are needed.

In the disease context, the cellular mechanisms observed in reactive astrocytes largely overlap with the experience‐dependent or physiological state‐driven changes in astrocytes. However, reactive astrocytes differ in the magnitude of the observed structural changes. In brain injury or neurodegeneration, astrocyte hypertrophy goes beyond a threshold that causes massive changes in gene expression profile, overexpression of GFAP and vimentin, and even disruption of astrocyte territories. Because of their relevance to disease, these cellular mechanisms have received much attention, but we are still far from understanding their function and utility. What is the threshold between physiological astrocyte plasticity and astrocyte reactivity? How can astrocytes regulate the magnitude of their structural responses? Addressing these unanswered questions may reveal new mechanisms regulating astrocyte structural plasticity. These insights have the potential to pinpoint the causes of and discover new treatments for brain diseases.

## CONFLICT OF INTEREST

The authors declare no potential conflict of interest.

## Data Availability

Data sharing is not applicable to this article as no new data were created or analyzed in this study.
